# Spontaneous Heterotopic Triplet Pregnancy With Tubal Rupture: A Case Report and Literature Review

**DOI:** 10.1177/2324709614531556

**Published:** 2014-04-16

**Authors:** Lima Arsala, Dennis Danso

**Affiliations:** 1 Royal Women’s Hospital, Melbourne, Victoria, Australia; 2 Latrobe Regional Hospital, Traralgon, Victoria, Australia

**Keywords:** heterotopic pregnancy, laparoscopy, ectopic pregnancy, transvaginal ultrasound

## Abstract

The recent increase in heterotopic pregnancies has been largely attributed to the increased use of assisted reproduction technologies. We report the rare case of a multiparous woman with a spontaneous conception resulting in a triplet heterotopic pregnancy: a twin intrauterine pregnancy and a single right tubal ectopic pregnancy. Heterotopic pregnancy is a rare and potentially life-threatening condition in which simultaneous gestations occur at 2 or more implantation sites. It is infrequent in natural conception cycles, occurring in 1:30 000 pregnancies. However, the prevalence is rising with the increased use of assisted reproduction techniques to that of 1:100 to 1:500 in these patient subgroups, highlighting the need to incorporate it into a clinician’s diagnostic algorithm.

Heterotopic pregnancy is a rare and potentially life-threatening condition in which simultaneous gestations occur at 2 or more implantation sites. It is infrequent in natural conception cycles, occurring in 1:30 000 pregnancies.^1,2^ However, the prevalence is rising with the increased use of assisted reproduction techniques to that of 1:100 to 1:500 in these patient subgroups, highlighting the need to incorporate it into a clinician’s diagnostic algorithm.^3^

## Case Report

**Figure 1.**
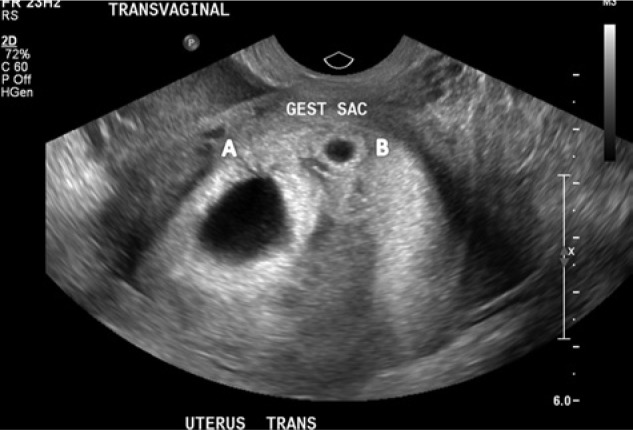
Transvaginal ultrasound of uterus showing 2 asymmetrical twin intrauterine gestational sacs. Fetuses A and B, both with cardiac activity, correspond to 6 weeks 3 days and 5 weeks and 6 days, respectively.

**Figure 2.**
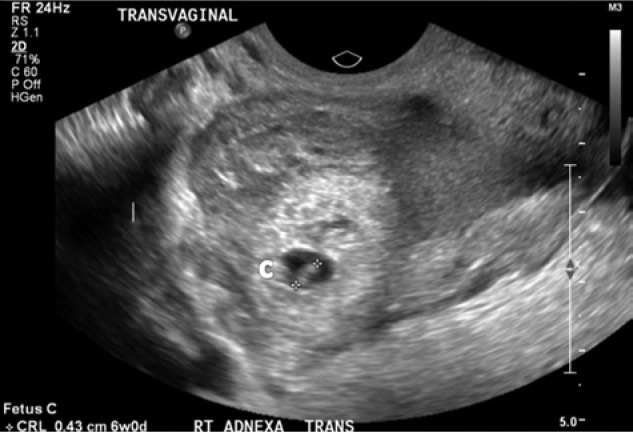
Transvaginal ultrasound of right adnexa showing the right tubal ectopic pregnancy (fetus C) with a gestational age of 6 weeks and cardiac activity.

A 27-year-old woman (gravida 5 para 2) presented to the emergency department with a complaint of sudden onset of right-sided lower abdominal pain in the setting of a recent positive home pregnancy test. Her last known menstrual period was 31 days prior to her presentation. She had no history of pelvic inflammatory disease, ectopic pregnancy, or fertility treatment. On physical examination, she was hemodynamically stable with a blood pressure of 110/60 mm Hg and heart rate of 82 beats/min. Abdominal examination demonstrated a distended abdomen with diffuse abdominal tenderness that was maximal in the right iliac fossa with rebound tenderness and signs of peritonism. Cervical tenderness was elicited during bimanual examination, with cervical excitation maximal in the right adnexa. Per speculum examination demonstrated a long, closed, posterior cervical os with no bleeding. Serum beta-hCG was 43 800 IU/L. Transvaginal sonography demonstrated 3 gestational sacs—2 intrauterine and 1 right adnexal—all with cardiac activity. The crown rump lengths (CRL) of the twin intrauterine fetuses were 6.7 mm and 3.6 mm corresponding to gestations of 6 weeks and 3 days and 5 weeks and 6 days, respectively (Figure 1). The right adnexal fetus had a CRL of 4.3 mm corresponding to a gestational age of 6 weeks (Figure 2). Ultrasonographic evidence of hemoperitoneum was present with a large amount of free fluid within the Pouch of Douglas.

**Figure 3.**
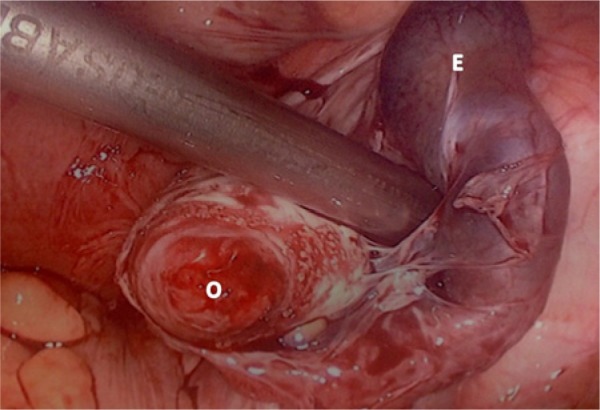
Laparoscopic image of right tubal ectopic gestation (E) with ovary (O) in view prior to removal and salpingectomy.

The patient underwent urgent laparoscopy that confirmed a ruptured right tubal ectopic pregnancy with hemoperitoneum (Figure 3). A laparoscopic right salpingectomy (using bipolar diathermy and scissors) was performed and effective hemostasis achieved. Hemoperitoneum was approximately 1.5 L, which was evacuated followed by peritoneal lavage. The histopathological examination of the excised right fallopian tube confirmed an ectopic pregnancy. The postoperative course was uneventful, with a hemoglobin level of 96 g/L, and the patient was discharged on postoperative day 1 on hematinics. Outpatient review with repeat ultrasound 8 days postoperatively demonstrated a single continuing viable intrauterine pregnancy with a CRL of 15.1 mm and a missed miscarriage of the second twin with a CRL static at 31 mm and an absent fetal heart. To date, the pregnancy is progressing well with ongoing antenatal care.

## Discussion

Heterotopic triplets in a natural cycle, with a tubal ectopic and coexisting twin intrauterine gestations, are very rare.^3,4^ With increased uptake of assisted reproduction techniques the overall incidence is increasing, occurring to up to 1:100 in these patient subgroups.^3,5^ It can encompass various clinical presentations including unilateral or bilateral tubal, cervical, abdominal, or ovarian pregnancies.^6,7^

Risk factors for heterotopic pregnancy are similar to those predisposing to ectopic pregnancies. These include previous tubal damage from pelvic inflammatory disease, endometriosis or tubal surgery, as well as previous ectopic pregnancy, cigarette smoking, in vitro fertilization, gamete intrafallopian transfer, and ovulation induction.^8-11^ Assessment of patients with the aforementioned risk factors should always highlight the potential of heterotopic pregnancy as a diagnosis. Nevertheless, the patient in our case report had no identifiable risk factors emphasizing the need to retain clinical vigilance to prevent overlooking rare diagnoses in the absence of risk factors.

The time of diagnosis of heterotopic pregnancy is quite variable, ranging from 5 to 34 weeks of gestation, with the majority of cases being diagnosed between 5 and 8 weeks of gestation.^1^ Diagnosis is fraught with difficulty due to the asymptomatic nature of the condition. Timely diagnosis allows conservative management options to be considered and allows surgical management to be planned.^12^ In the case of tubal heterotopic pregnancy, diagnosis is often made after tubal rupture and presentation with an acute abdomen, with few cases reported having been diagnosed prior to this.^4^ Of note, vaginal bleeding is commonly absent in the clinical setting of heterotopic pregnancy presenting as tubal ectopic pregnancy, adding to the complexity of clinical diagnosis.^5^

The principal diagnostic aid is transvaginal sonography (TVS), with a recent study by Li et al^12^ demonstrating 92.4% sensitivity and 100% specificity for the detection of heterotopic pregnancy in their patient cohort. However, there are conflicting reports with previous studies demonstrating lower sensitivities with TVS for detection of ectopic or heterotopic pregnancy. Some studies quote detection rates ranging between 41% and 84%. Systematic and repeated TVS studies may increase the detection rate.^5,11,13^ Low sonographic detection rates of heterotopic pregnancy may be attributed to false reassurance given by the sonographic confirmation of an intrauterine pregnancy and subsequent limited sonographic adnexal examination for additional gestational sacs.^1,5^ Furthermore, there is the added difficulty of differentiating between an anembryonic adnexal gestation sac and a hemorrhagic corpus luteal cyst, and repeat ultrasound to look for interval growth will confirm the diagnosis.^14^ Ultrasonographic features that may increase detection of heterotopic pregnancy include an extrauterine gestational sac with fetal cardiac activity, fetal node, hyperechogenic ring surrounding the gestational sac, and an adnexal mass.^8^ Clinicians should routinely consider early TVS in those women with known risk factors for heterotopic pregnancy to confirm pregnancy location. Serial serum β-hCG testing to look for the rapid rise in early pregnancy tends to be misleading in the diagnosis of heterotopic pregnancy as subnormal hormone production from the ectopic gestation may potentially be masked by the higher placental production from an intrauterine pregnancy and this cannot be relied upon.^3,15^

Once diagnosis of heterotopic pregnancy has been made, the management is primarily surgical, although other modalities have been reported in the literature. First, there is the option in some cases for conservative management with spontaneous resolution.^16^ However, this approach is fraught with danger due to the unstable nature of extrauterine gestations with the potential of rupture resulting in maternal hemodynamic compromise and jeopardy of intrauterine gestation(s). Adding to this is the lack of clear guidelines as to which patients are able to be safely managed expectantly and how they are best assessed for interval gestational growth and heterotopic pregnancy resolution. Medical management encompasses laparoscopic or TVS-guided injection of potassium chloride or hyperosmolar glucose into an intact ectopic or heterotopic gestational sac.^17^ However, over half of those with tubal heterotopic gestations treated with this modality require subsequent salpingectomy, raising concerns about the effectiveness of such treatment for this patient subset.^18^ It may be most of use in unusual extrauterine locations that are not as easily amenable to surgical approaches, such as cervical or caesarean section scar heterotopic pregnancies.^19,20^ Other medical treatments, such as methotrexate or prostaglandins, used in the management of ectopic pregnancies are not suitable for use in heterotopic pregnancies due to adverse effects on concurrent intrauterine gestations.^21^

The gold standard in management of heterotopic pregnancies is surgery via laparoscopy or laparotomy, with the surgical approach guided by the clinical scenario.^1-3^ Laparoscopic approaches are preferred to open procedures except in cases of clinical shock with intra-abdominal hemorrhage where laparotomy may be the better suited procedure.^1^ In our case report, we opted for laparoscopic salpingectomy as the patient was hemodynamically stable and amenable to a laparoscopic versus an open surgical approach. Current evidence shows that despite our best efforts, the intrauterine component of a heterotopic pregnancy has a higher likelihood of miscarriage than sole intrauterine pregnancies, although survival rates of intrauterine heterotopic pregnancies have improved over the past few decades with those that proceed to live birth demonstrating no significantly different rates of adverse birth outcomes.^21,22^

## Conclusion

Heterotopic pregnancy is a potentially life-threatening condition that, while being rare, potentially has grave implications for both the mother and fetus. High-risk groups warrant early pregnancy ultrasound as a part of routine antenatal care to enable early diagnosis and timely management.^12^ However, an absence of risk factors should not equate to exclusion. As highlighted in our case above, it must remain at the forefront of a clinician’s diagnostic algorithm in all women as it may occur in the absence of risk factors in a natural conception cycle.^1,2^ Despite it being a challenging diagnosis, clinical acumen along with skilled TVS and timely management is able to achieve optimal clinical outcomes.^1,12^
